# Quality of life in children with erythropoietic protoporphyria: a case–control study

**DOI:** 10.1111/1346-8138.17348

**Published:** 2024-06-26

**Authors:** Louisa G. Kluijver, Debby Wensink, Margreet A. E. M. Wagenmakers, Hidde H. Huidekoper, Peter Witters, Daisy Rymen, Janneke G. Langendonk

**Affiliations:** ^1^ Department of Internal Medicine, Porphyria Center Rotterdam, Center for Lysosomal and Metabolic Diseases, Erasmus MC University Medical Center Rotterdam The Netherlands; ^2^ Department of Pediatrics, Center for Lysosomal and Metabolic Diseases, Erasmus MC University Medical Center Rotterdam The Netherlands; ^3^ Department of Pediatrics, Center for Metabolic Diseases University Hospitals Leuven Leuven Belgium; ^4^ Department of Development and Regeneration KU Leuven Leuven Belgium

**Keywords:** children, erythropoietic protoporphyria, photosensitivity disorder, quality of life, social participation

## Abstract

Erythropoietic protoporphyria (EPP) is an inherited metabolic disease that causes painful phototoxic reactions, starting in childhood. Studies have shown a reduced quality of life (QoL) in adults with EPP, however, data on children with the disease are lacking. Since treatment for EPP is currently not registered for children, knowledge about their QoL is of crucial importance. In this prospective, case–control study, we included children from the Netherlands and Belgium diagnosed with EPP and matched to healthy controls. Previously collected EPP quality of life (EPP‐QoL) data from matched adults with EPP were used. QoL scores, utilizing the Pediatric Quality of Life Inventory (PedsQL) and the disease‐specific EPP‐QoL, were collected. Scores range from 0 to 100, with higher scores indicating a higher QoL. Non‐parametric tests were used to compare groups. A total of 15 cases, 13 matched healthy control children, and 15 matched adults with EPP were included. Children with EPP exhibited lower median scores in the PedsQL in both physical (cases: 87.5 (interquartile range [IQR] 77.7–96.1), controls: 99.2 [IQR 94.9–100.0], *p* = 0.03) and social (cases: 77.5 [IQR 69.4–86.3], controls: 97.5 [IQR 78.8–100.0], *p* = 0.04) domains compared to healthy children, although these differences were not statistically significant after correcting for multiple testing. The overall median EPP‐QoL score for children was similar to adults with EPP (children: 44.4 [IQR 25.0–54.2], adults: 45.8 [IQR 25.7–68.1], *p* = 0.68). However, within the EPP‐QoL subdomain on QoL, children were found to have significantly lower median scores (children: 16.7 [IQR 0.0–33.3], adults: 33.3 [IQR 33.3–62.5], *p* < 0.01). In conclusion, children with EPP experience a reduced QoL compared to both healthy children and adults with EPP. Ensuring treatment availability for this patient group is crucial for improving their QoL. We advocate the inclusion of children in safety and efficacy studies, to ensure availability of treatment in the future.

## 1 INTRODUCTION

Erythropoietic protoporphyria (EPP; OMIM 177000) is an inherited metabolic disease caused by the deficiency of ferrochelatase, the terminal enzyme in the heme biosynthesis pathway, resulting in phototoxic reactions due to the accumulation of protoporphyrin IX. These phototoxic reactions, which start from early childhood, cause severe pain, which results in lifelong light‐avoiding behavior that limits daily and social activities. Phototoxic reactions can occur after only a few minutes of sunlight exposure and can last up to a week.^1^ The pain is untreatable as it does not respond to analgesics.

Quality of life (QoL) in adult patients with EPP is, therefore, low. This was established in prior research using the disease‐specific quality of life questionnaire (EPP‐QoL),^2^ which found that QoL fell within a range of 30%–44%.^3^, ^4^, ^5^, ^6^ EPP significantly affects both physical functioning, due to intense untreatable pain, and social functioning.^3^ Adults with EPP have also reported that their highest burden of disease was during their childhood,^3^, ^7^, ^8^ but the QoL of children with EPP has never been investigated.

Since 2016, afamelanotide has been authorized by the European Medicines Agency for the treatment of adult patients with EPP, and since 2019, it has also been authorized by the US Food and Drug Administration. Afamelanotide is an α‐melanocyte‐stimulating hormone analogue that stimulates eumelanin production, thereby reducing free radical production.^5^ The QoL of adults with EPP is reported to improve significantly with afamelanotide treatment.^3^, ^4^, ^5^, ^6^ These findings highlight the importance and need for treatment options for children with EPP. However, as the safety of afamelanotide has not been investigated in children, the treatment is currently unavailable for them, leaving them to a life mainly confined to indoors.

Acknowledging the high burden of disease and the current unavailability of treatment, investigating the QoL of children with EPP is essential. In the present study, our aim was to compare QoL in children with EPP to that in healthy children and adult patients with EPP. We hypothesized that children with EPP have a reduced QoL when compared to their healthy peers and to adults with EPP.

## 2 METHODS

In this prospective, case–control study, we included children diagnosed with EPP and healthy matched controls. Participants were recruited during the summer, from May 2020 to September 2022. Children aged 8 to 18 years with EPP, followed up at the Erasmus MC Sophia Children's Hospital the Netherlands, the Isala Clinic in Zwolle, the Netherlands, or the University Hospitals Leuven in Belgium, were eligible for inclusion. Diagnosis of EPP was based on clinical characteristics and elevated protoporphyrin IX levels (>4 times upper limit of normal) and/or *FECH* mutation analysis. Patients with other conditions that could affect their QoL were excluded.

Healthy control children were family, friends, or acquaintances of the EPP participant. They were matched 1:1 based on age (±2 years), sex, education (primary or secondary school), and nationality. Exclusion criteria for controls were any photosensitivity, family members who lived within the same household as an EPP patient, and/or another condition that might limit their daily activities or QoL.

Children with EPP were also compared to adults with EPP, to assess disease‐specific QoL. All adults with EPP completed a questionnaire during routine visits to the outpatient clinic before treatment with afamelanotide, between August 2014 and January 2018. From the adult patient database, adults with EPP were matched 1:1 with children based on sex, self‐reported age at time of EPP diagnosis (± 5 years), and self‐reported disease severity, measured by minutes spent in direct sunlight until first symptoms occur (± 5 minutes).

Participants characteristics and QoL scores were collected during a one‐time assessment. This included three questionnaires including two QoL questionnaires, the validated Pediatric Quality of Life inventory (PedsQL), and EPP‐QoL questionnaire. Lastly, the validated type D scale‐14 (DS‐14) questionnaire, which assesses negative affectivity and social inhibition, was used. While the EPP‐QoL is validated, it was designed for adult use and has not been validated for children. Other questions on patient characteristics, medication use, medical history, school participation, vacations, and a visual analog scale (VAS) for perceived health (scores from 0–100) were collected. Protoporphyrin IX levels measured around the time of the QoL assessment (± 1 year) for both adults and children with EPP were recorded. Additionally, data on FECH genotype and variant types were collected.

To compare quality of life between EPP cases and controls, the validated Dutch translated PedsQL, version 4.0 (the version enquiring over the past month) was utilized.^9^, ^10^ The questionnaire included both children's reports and parent proxy reports on the child's QoL. Two different versions were used, depending on the age of participant: children aged 8–12 or teenagers aged 13–18 years. A total of 23 questions were scored on a 5‐point Likert scale from 0 to 100, higher scores indicating a higher QoL. Questions cover four different domains of QoL including; physical (eight questions), emotional (five questions), social (five questions), and school functioning (five questions). Psychosocial scores were the sum of emotional, social, and school functioning. Total scores included all four domains.

To compare quality of life between children and adult EPP patients, the disease‐specific EPP‐QoL questionnaire was used.^2^ The revised version includes a total of 12 questions covering two domains: severity of disease, containing a total of 10 questions, and QoL, containing two questions. Questions were scored on a 4‐point Likert scale with scores of 0 to 3 for positive statements and 3 to 0 for negative statements. Responses were then transformed into a 0–100 scale, higher scores indicating a higher quality of life.

Lastly, we used the DS‐14 questionnaire, which consists of 14 questions:seven on negative affectivity and seven on social inhibition.^11^ Negative affectivity refers to the tendency to experience negative emotions. High negative affectivity individuals experience more feelings of dysphoria, anxiety, and irritability. They have a negative view of self and scan their environment for signs of impending trouble. Social inhibition refers to the tendency to inhibit the expression of emotions and behaviors in social interactions, to avoid disapproval by others. High social inhibition individuals tend to feel inhibited, tense, and insecure when with others. Questions were rated on a 5‐point Likert Scale ranging from 0 to 4. The total score for each domain (social inhibition or negative affectivity) was 28. If scores equaled 10 or more on either domain they were regarded as positive for social inhibition or negative affectivity.^11^

Data on children with EPP and healthy controls was entered and stored in Castor EDC, version 2022.5.2.0, from 2021 to 2023. A database validation check was performed in 10% of all data, chosen at random by a fellow investigator. An error percentage of <5% was considered acceptable. Previously collected data on adults with EPP were exported from the database program, OpenClinica open source software, version 2.1.

For analyses, data were summarized using median and interquartile range for continuous non‐parametric data, and frequencies and percentages for categorical data. In instances where all cases had matched controls, paired non‐parametric tests were employed. In instances where complete matching was not achieved, unpaired non‐parametric tests were used. Statistical tests with *p*‐values were 2‐sided with a significance level of 0.05. For the analysis of the PedsQL and EPP‐QoL questionnaires, the *p*‐values were corrected for multiple testing using the Bonferroni correction. Missing values within the PedsQL and EPP‐QoL were imputed by the median value if more than 50% of the questionnaire was filled in. For PedsQL, this included a total of two values and for EPP‐QoL five values. All statistical analysis were performed using R Studio, version 2021.09.2.

## 3 RESULTS

A total of 15 children with EPP were included with 13 matched healthy children as controls. Two children were unable to provide a healthy control. A total of nine children with EPP were matched on age, sex, education, and nationality. The remaining four did not match completely; one based on education, three based on sex, of whom one also did not match on nationality. One control and three children with EPP were excluded from the PedsQL analysis: two due to missing data, and two due to the presence of additional conditions that limited their daily activities, these included pulmonary stenosis and asthma. The child with EPP with an additional condition was included in the EPP‐QoL analysis as the questions are disease‐specific, and the presence of additional conditions would not significantly impact the responses. For the adult controls, 15 individuals were successfully matched on sex, 13 on age of diagnosis, and 12 on disease severity.

Baseline characteristics of participants are presented in Table 1, which includes children with EPP as cases and both healthy children and adults with EPP as controls. The proportion of male participants was lower in the children with EPP compared to healthy children (cases: 20%, controls: 46%, *p* = 0.07). Median (IQR) age was similar across child groups (cases: 13.0 [11.0–15.0], controls: 12.0 [11.0–15.0], *p* = 0.85). A significantly higher proportion of children with EPP attended secondary school compared to healthy children (cases: 60.0%, controls: 46.2%, *p* < 0.01). Most healthy children from the control group were friends of the cases (76.9%), some knew each other from extracurricular activities (15.4%), and one control was a family member who did not reside within the same household (7.7%).

**TABLE 1:** Baseline characteristics.

	Cases	Controls			
	Children with EPP	Matched healthy children	*p* ^a^	Matched adults with EPP	*p* ^b^
Participants, *n*	15	13		15	
Sex, male, %	20.0	46.2	0.07	20.0	1.00
Age, years	13.0 (11.0–15.0)	12.0 (11.0–15.0)	0.85	42.0 (31.5–50.0)	<0.001*
Age diagnosis, years	5.0 (4.0–6.8)	n.a.	n.a.	6.0 (2.0–8.0)	0.57
Missing, %	6.7			0.0	
Fitzpatrick skin type, %			1.00		1.00
Type I	13.3	0.0		13.3	
Type II	53.3	30.8		53.3	
Type III	20.0	61.5		20.0	
Type IV	0.0	0.0		0.0	
Type V	0.0	0.0		0.0	
Missing, %	13.3	7.7		13.3	
Education, %			<0.01*	n.a.	n.a.
Primary school	40.0	53.8			
Secondary school	60.0	46.2			
Secondary school, %^c^	60.0	46.2	0.10		
Lower level	33.3	23.1			
Higher level	26.7	23.1			
Country of birth, %			0.04*		0.13
Netherlands	86.7	76.9		93.3	
Belgium	13.3	15.4		0.0	
USA	0.0	7.7		6.7	
Nationality, %			0.15		0.13
Dutch	93.3	74.6		100.0	
Belgian	6.7	7.7		0.0	
Moroccan	0.0	7.7		0.0	
Relationship with case, %	n.a.		n.a.	n.a.	n.a.
Family^d^		7.7			
Friends		76.9			
School or sports		15.4			
Photosensitivity (min)^e^	22.5 (5.0–30.0)	^f^	n.a.	15.0 (5.0–45.0)	0.28
Missing (%)	6.7	7.7		0.0	
Protoporphyrin IX (μmol/L erythrocytes)	33.0 (20.0–52.0)	n.a.	n.a.	45.0 (28.4–88.8)	0.27
Missing, %	13.3			0.0	
FECH genotype, %		n.a.	n.a.		1.00
FECH variant and Polymorphism (c.315‐48T>C)	93.3			93.3	
Missing, %	6.7			6.7	
FECH variant type, %		n.a.	n.a.		0.97
Missense	46.7			40.0	
Nonsense	0.0			26.7	
Splice‐site	33.3			20.0	
Deletion	6.7			6.7	
Deletion and insertion	6.7			0.0	
Missing (%)	6.7			6.7	

*Note*: Data is given in percentages for categorical values or median (interquartile range) for continuous variables. All significant *p*‐values are bold and noted by an asterisk (*).

Abbreviations: EPP, erythropoietic protoporphyria; FECH, ferrochelatase; n.a., not applicable.

^a^ *p*‐values were calculated using Fisher's exact test for categorical and Mann–Whitney *U*‐test for continuous variables.

^b^ *p*‐values were calculated using the McNemar test for categorical values or Wilcoxon signed‐ranked test for continuous values.

^c^ Secondary school was divided into the lower level, including preparatory secondary vocational education (in Dutch: VMBO, Voorbereidend Middelbaar Beroepsonderwijs), and higher level, including both higher general secondary education (in Dutch: HAVO, Hoger Algemeen Voortgezet Onderwijs), and preparatory scientific education (in Dutch: VWO, Voorbereidend Wetenschappelijke Onderwijs).

^d^ Family living in the same household were excluded.

^e^ This was measured using the following questions. For patients with EPP: “How long can you be outside in direct sunlight before you experience EPP symptoms?” For matched healthy children: “How long can you be outside in direct sunlight?”

^f^ All reported unlimited.

The proportion of males was the same in children with EPP compared to adults with EPP (children: 20%, adults: 20%, *p* = 1.00). They exhibited a similar age at diagnosis (children: 5.0 [IQR 4.0–6.8], adults: 6.0 [IQR 2.0–8.0], *p* = 0.57). The severity of photosensitivity, estimated as the reported time spent outside in direct sunlight until symptoms manifested, was similar across groups (children: 22.5 minutes [IQR 5.0–30.0], adults: 15.0 [IQR 5.0–45.0], *p* = 0.28).

Characteristics on school, career, and vacation participation are shown in Table 2. Nearly half of the children with EPP (46.6%) reported having restrictions at school, while only 13% of healthy controls reported restrictions (*p* = 0.07). Furthermore, all children (100.0%) with EPP needed adjustments at school to enable their participation, compared to none (0.0%) of the healthy children (*p* < 0.001). Most (60.0%) mentioned being restricted in their career choices because of EPP, compared 6.7% of the healthy controls (*p* = 0.07). Almost all children with EPP reported being restricted in their vacations (cases: 93.3%, controls: 0.0%, *p* < 0.01), with 53.3% needing to adjust their destination, 33.3% their activities, and 6.7% reporting not being able to go on vacation because of EPP.

**TABLE 2:** Characteristics of children with EPP and matched healthy children.

	Children with EPP (cases)	Matched healthy children (controls)	*p*
Participants, *n*	15	13	
School participation, %			0.07
Unrestricted daily attendance	40.0	66.7	
Cannot attend everyday due to health conditions	13.3	0.0	
Other^a^	33.3	13.3	
Missing, %	13.3	7.7	
Restrictions in making career choice/choice affected by EPP	60.0	6.7	0.07
Missing	6.7	23.1	
Adjustments at school are needed	100.0	0.0	<0.001*
Vacation			<0.01*
Unrestricted	6.7	100.0	
Restricted	93.3	0.0	
Vacation restrictions	93.3	0.0	1.00
Need to adjust destination	53.3	0.0	
Need to adjust activities	33.3	0.0	
I cannot go on vacation due to EPP	6.7	0.0	
I cannot go on vacation due to other reasons	0.0	0.0	
Type of vacation			0.49
Beach	6.7	46.2	
Active	13.3	38.5	
Camping	13.3	15.4	
City trip	6.7	0.0	
Forrest	46.7	0.0	
Other^b^	6.7	0.0	
Missing	6.7	0.0	

*Note*: All data is given in percentages (categorical values). All *p*‐values were calculated using the Fisher's exact test. All significant *p*‐values are bold and noted by an asterisk (*).

Abbreviations: EPP, erythropoietic protoporphyria.

^a^ Other case responses: “I stay at home with warm weathers”, “I can go to school with adjustments”, “I am restricted depending on what month, if it is really warm weather or sunny outside I need to stay inside during recess”, “I go to school daily but I am restricted due to EPP”. Controls, “I have 1 day of school and 5 days of work”, “I go to primary school”.

^b^ Other: cases, holiday home/cottage.

**FIGURE 1:**
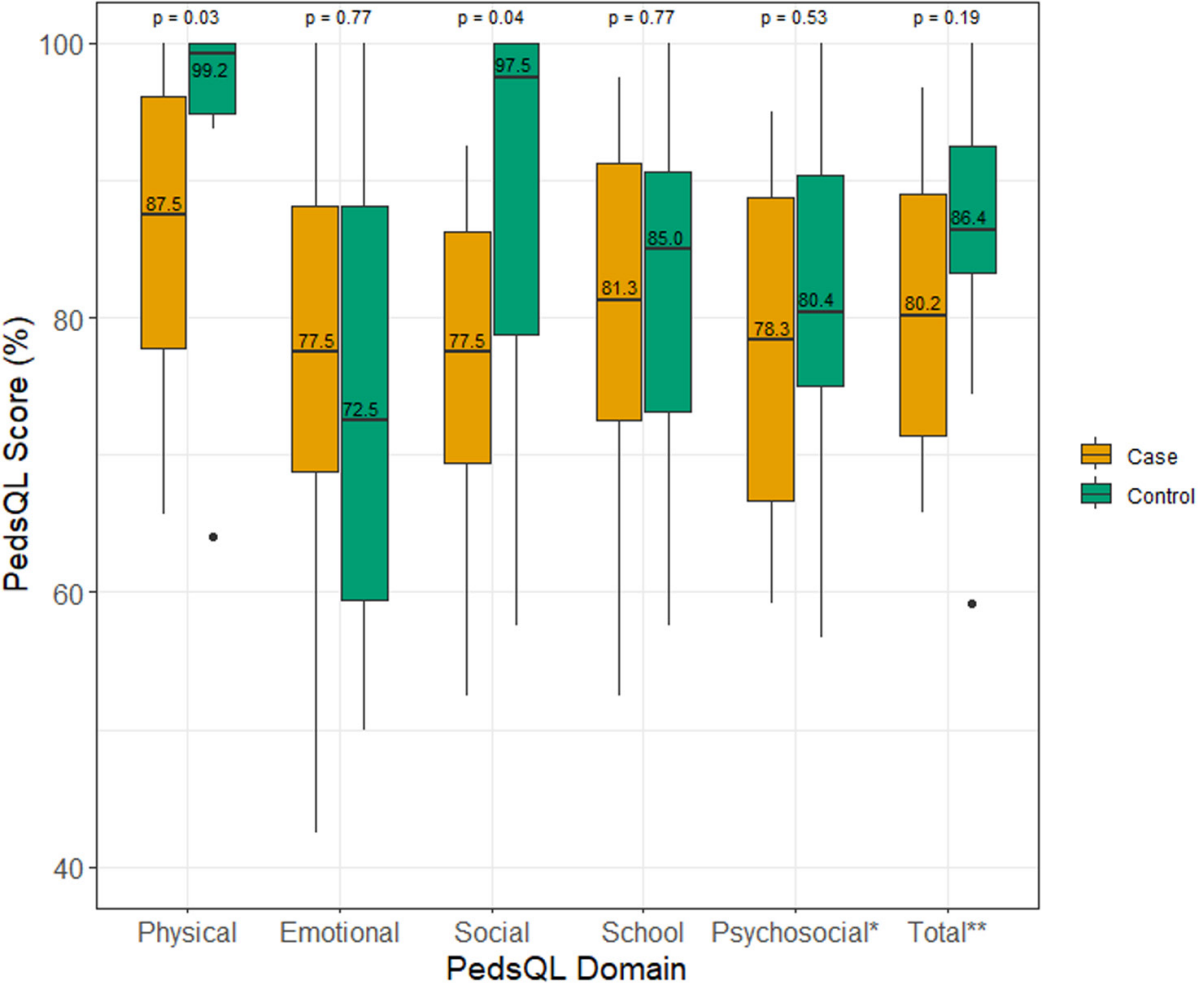
Quality of life scores based on the Pediatric Quality of Life Inventory (PedsQL) of children with erythropoietic protoporphyria (EPP; cases) compared to matched healthy children (controls). The figure illustrates the median scores and interquartile range of the children with EPP in orange (case, left) and matched healthy children in green (control, right). The median value is indicated inside each box, with *p*‐values from Mann–Whitney *U* tests for each domain included in the figure. Significant *p*‐values are indicated in bold and marked with an asterisk (*). **includes emotional, social and school functioning. ***includes physical, emotional, social, and school functioning.

In Table 3 and Figure 1, we present median (IQR) scores on QoL from the PedsQL questionnaire. Children with EPP exhibited lower scores in the physical functioning domain compared to healthy controls (cases: 87.5 [IQR 77.7–96.1], controls: 99.2 [IQR 94.9–100.0], *p* = 0.03). This difference was particularly evident in the areas of sports participation (cases: 87.5 [IQR 68.8–100.0], controls: 100.0 [IQR 100.0–100.0], *p* = 0.03); pain (cases: 68.8 [IQR 50.0–81.3], controls: 100.0 [IQR 75.0–100.0], *p* = 0.03); and low energy (cases: 75.0 [IQR 71.9–100.0], controls: 100.0 [IQR 87.5–100.0], *p* = 0.03). These differences were not statistically significant after correcting for multiple testing.

**TABLE 3:** Quality of life based on PedsQL scores of children with EPP and matched healthy children.

	Children with EPP (cases)	Matched healthy children (controls)	*p*
Participants, *n*	12	12	
Physical functioning	87.5 (77.7–96.1)	99.2 (94.9–100.0)	0.03
Walking	100.0 (84.4–91.7)	100.0 (100.0–100.0)	0.11
Running	100.0 (87.5–100.0)	100.0 (100.0–100.0)	0.18
Sports participation	87.5 (68.8–100.0)	100.0 (100.0–100.0)	0.03
Lifting heavy	100.0 (84.4–100.0)	100.0 (100.0–100.0)	0.40
Showering by myself	100.0 (100.0–100.0)	100.0 (100.0–100.0)	0.36.
Chores around the house	100.0 (93.8–100.0)	100.0 (100.0–100.0)	0.33
Pain, Wounds	68.8 (50.0–81.3)	100.0 (75.0–100.0)	0.03
Low energy	75 (71.9–100.0)	100.0 (87.5–100.0)	0.03
Emotional functioning	77.5 (68.8–88.1)	72.5 (59.4–88.1)	0.77
Afraid/scared	87.5 (75.0–100.0)	68.8 (62.5–90.6)	0.18
Sad/blue	75 (62.5–90.6)	68.8 (62.5–90.6)	0.70
Angry	75 (71.2–87.5)	68.8 (59.4–81.3)	0.37
Trouble sleeping	62.5 (46.9–90.6)	81.3 (59.4–100.0)	0.30
Worrying	87.5 (71.9–100.0)	75 (62.5–90.6)	0.60
Social functioning	77.5 (69.4–86.3)	97.5 (78.8–100.0)	0.04
Getting along with kids	87.5 (75.0–100.0)	100.0 (84.4–100.0)	0.54
Kids wanting to be my friend	100.0 (87.5–100.0)	93.8 (68.8–100.0)	0.51
Teasing	100.0 (96.9–100.0)	100.0 (75.0–100.0)	0.64
Cannot do things other kids do	75.0 (43.8–75.0)	100.0 (84.4–100.0)	0.01*
Hard to keep up when playing with other kids	50.0 (37.5–65.6)	100.0 (96.9–100)	<0.001*
School functioning	81.3 (72.5–91.3)	85.0 (73.1–90.6)	0.77
Paying attention	81.3 (62.5–100.0)	75.0 (62.5–100.0)	0.79
Forgetting things	81.3 (62.5–100.0)	75.0 (59.4–78.1)	0.34
Keeping up with schoolwork	87.5 (75.0–100.0)	75.0 (75.0–100.0)	0.47
Missing school	81.3 (75.0–100.0)	100.0 (75.0–100.0)	0.55
Missing school due to hospital visits	75.0 (62.5–78.1)	100.0 (96.9–100.0)	0.02
Psychosocial score^a^	78.3 (66.7–88.8)	80.4 (75.0–90.4)	0.53
Total score^b^	80.2 (71.5–89.0)	86.4 (83.3–92.5)	0.19

*Note*: All data are given as median (interquartile range). Scores range from 0 to 100, with higher scores indicating a higher quality of life. *p*‐values are calculated using the Mann–Whitney *U*‐test. All *p*‐values <0.05 are bold, and all significant *p*‐values after Bonferroni correction (0.05/4 = 0.0125 are noted by an asterisk (*)).

Abbreviation: EPP, erythropoietic protoporphyria; PedsQL, Pediatric Quality of Life Inventory.

^a^ Includes emotional, social, and school functioning.

^b^ Includes physical, emotional, social, and school functioning.

Patients with EPP also scored lower in social functioning (cases: 77.5 [IQR 69.4–86.3], controls: 97.5 [IQR 78.8–100.0], *p* = 0.04). Specifically, statistically significant differences were observed in responses to “Cannot do things other kids do” (cases: 75.0 [IQR 43.8–75.0], controls: 100.0 [IQR 84.4–100.0], *p* = 0.01) and “Hard to keep up when playing with other kids” (cases: 50.0 [IQR 37.5–65.5], controls: 100.0 [IQR 96.9–100.0], *p* < 0.001).

Patients with EPP reported lower scores on “missing school due to hospital visits” (cases: 75.0 [IQR 62.5–78.1], controls: 100.0 [IQR 96.9–100.0], *p* = 0.02) within the school domain, although this difference was not statistically significant after correcting for multiple testing. Other than that, EPP patients scored comparably to the healthy controls with respect to school functioning. No significant differences were observed between EPP patients and controls in the emotional functioning, psychosocial scores, and total scores. Table S1 presents a paired analysis of the PedsQL, revealing consistent findings. Stratified analysis based on age, sex, year of inclusion, and child versus parent‐proxy reports are is shown in Tables S2–S5.

In Table 4, PedsQL scores from children with EPP are compared to children from the Dutch reference population.^12^ Scores were divided between children aged 8 to 12 and teenagers aged 13 to 18 years; means were used for comparison. Although not statistically tested, children seemed to have scored lower in all domains when compared to the healthy reference population, while teenagers only scored lower in social functioning. Notably, our matched healthy children seemed to have scored higher than the Dutch reference population.

**TABLE 4:** Comparing Quality of life based on the PedsQL of children with EPP, matched controls, and a Dutch children reference Population.

	Children with EPP (cases)	Matched healthy children (controls)	Healthy reference population NL^12^
Children (8–12 years)			
Participants, *n*	5	8	192
Physical functioning	84.4 (9.2)	98.8 (2.2)	85.3 (8.8)
Emotional functioning	70.0 (16.5)	73.1 (18.1)	76.9 (13.8)
Social functioning	76.5 (15.7)	92.8 (10.5)	76.5 (12.2)
School functioning	78.0 (16.3)	83.8 (12.2)	78.9 (11.9)
Psychosocial score^a^	74.8 (13.3)	83.2 (9.8)	80.8 (10.3)
Total score^b^	78.2 (9.1)	88.7 (6.5)	82.3 (8.8)
Teenagers (13–18 years)			
Participants, *n*	7	4	148
Physical functioning	87.5 (13.2)	88.3 (16.4)	86.8 (9.2)
Emotional functioning	81.4 (17.6)	78.1 (19.1)	77.5 (15)
Social functioning	76.8 (11.3)	78.8 (21.8)	90.1 (11.4)
School functioning	82.1 (11.7)	75.0 (21.1)	76.0 (12.7)
Psychosocial score^a^	80.1 (12.9)	77.3 (20.3)	81.2 (10.2)
Total score^b^	82.7 (82.7)	81.1 (18.0)	83.1 (8.9)

*Note*: Data is given in mean (standard deviation) for comparisons to prior research.^12^ Scores range from 0 to 100, with higher scores indicating a higher quality of life.

Abbreviations: EPP, erythropoietic protoporphyria; NL, the Netherlands. PEDsQL, Pediatric Quality of Life Inventory.

^a^ Includes emotional, social and school functioning.

^b^ Includes physical, emotional, social and school functioning.

**FIGURE 2:**
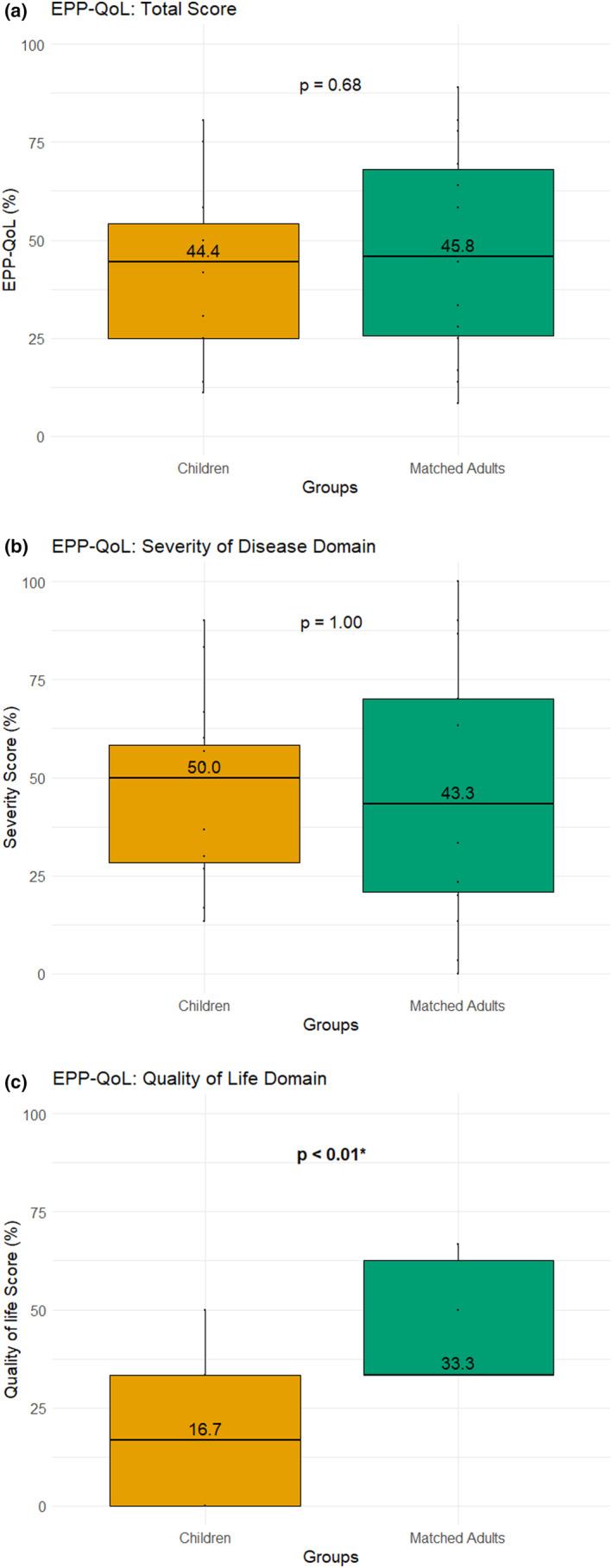
Erythropoietic protoporphyria (EPP) specific Quality of life Questionnaire (EPP‐QoL) of children with EPP (cases) compared to adult with EPP (controls). The figure illustrates the median scores and interquartile range of the children in orange (left) and matched adults with EPP in green (right). The median value is indicated inside each box, with *p*‐values from Wilcoxon signed‐rank tests included in the figure. Significant *p*‐values are indicated in bold and marked with an asterisk (*). The figure presents data for three categories: (a) Total EPP‐QoL Score, (b) Severity of Disease Domain Score, and (c) Quality of Life Domain Score.

Table 5 and Figure 2 present the outcomes for the EPP‐QoL scores. The median QoL score for children with EPP closely resembled the QoL scores of matched adults with EPP prior to treatment with afamelanotide (children: 44.4% [IQR 25.0–54.2], adults: 45.8%, [IQR 25.7–68.1], *p* = 0.68). Furthermore, the QoL scores among children in our study aligned with previously reported EPP‐QoL scores in untreated adult EPP populations, which were found to be at 44.0% (IQR 21.2–69.4).^3^

**TABLE 5:** EPP‐QoL questionnaire scores of children with EPP compared to adults with EPP.

	Children with EPP (cases)	Matched adults with EPP (controls)	*p*	Prior research	
				Adults with EPP before^a^ ^,^ ^3^	Adults with EPP treated^b^ ^,^ ^3^
Number of participants, *n*	15	15		119	100
Total score	44.4 (25.0–54.2)	45.8 (25.7–68.1)	0.68	44.0 (21.2–69.4)	75.0 (63.9–88.9)
Missing	0.0	6.7			
Quality of life Domain	16.7 (0.0–33.3)	33.3 (33.3–62.5)	<0.01*	n.a.	n.a.
Severity of disease Domain	50.0 (28.3–58.3)	43.3 (20.8–70.0)	1.00	n.a.	n.a.

*Note*: All data is given in median, and first and third quartile for comparisons to prior research.^3^ *p*‐values are calculated using Wilcoxon signed‐rank tests. All significant *p*‐values after Bonferroni correction (0.05/2 = 0.025) are bold and noted by an asterisk (*). Scores range from 0 to 100, with higher scores indicating a higher QoL.

Abbreviation: EPP, erythropoietic protoporphyria. EPP‐QoL, Erythropoietic protoporphyria Quality of Life questionnaire.

^a^ “Adults with EPP before” refers to adults with EPP before they initiated treatment with afamelanotide.

^b^ Adults treated with EPP refers to the scores of adults with EPP who were treated with afamelanotide.

When we further split the total EPP‐QoL score into its two domains, quality of life and disease severity, a different trend became evident. We observed significantly lower median scores in the QoL domain in children with EPP (children: 16.7% [IQR 0.0–33.3], adults: 33.3% [IQR 33.3–62.5], *p* < 0.01). In contrast, no significant difference was detected in the disease severity scores (children: 50.0 [IQR 28.3–58.33], adults: 43.3 [IQR 20.8–70.0], *p* = 0.97).

Outcomes on the VAS health perception score and the DS‐14 questionnaire on negative affectivity and social inhibition scores are shown in Table 6 and Figure S1. The scores of children with EPP were comparable to adults with EPP from a prior study.^3^ Children with EPP exhibited significantly more social inhibition compared to healthy children (cases: 66.7%, controls: 46.2%, *p* = 0.02). There was no difference found in negative affectivity between cases and controls. The median VAS health perception scores were significantly lower in children with EPP compared to healthy children (cases: 80.0 [IQR 78.5–90.0] controls: 91.0 [IQR 90.0–97.0], *p* = 0.04).

**TABLE 6:** Differences in VAS health perception scores, social inhibition, and negative affectivity of children with EPP, healthy matched children, and adults with EPP.

	Children with EPP (cases)	Matched healthy children (controls)	*p*	Adults with EPP Before^3^	Adults with EPP Treated^3^
Participants (*n*)	15	13	n.a.	95	95
Social inhibition^a^	66.7	46.2	0.02*	41.1	34.7
Social inhibition score	12.0 (5.5–15.5)	8 (3.0–11.0)	0.31	7.0 (3.0–15.0)	7.0 (2.0–11.0)
Negative affectivity^a^ (%)	40.0	46.2	0.59	42.1	40.0
Negative affectivity score	7.0 (5.0–12.0)	8 (4.0–13.0)	0.96	8.0 (4.0–13.0)	7.0 (2.0–13.0)
Health perception score	80.0 (78.5–90)	91.0 (90.0–97.0)	0.04*	80.0 (70–90)	80 (70–90)

*Note*: All data is given in percentages for categorical values or median (first and third quartile) for continuous variables and compared to prior research.^3^ *p*‐values are calculated using Mann Whitney *U*‐test for continuous values and Fisher's exact test for categorical values. All significant *p*‐values are bold and noted by an asterisk (*).

Abbreviation: EPP, erythropoietic protoporphyria; n.a., not applicable; VAS, visual analog scale.

^a^ Social inhibition and negative affectivity is defined as on a score ≥ 10.

Lastly, our analysis revealed no significant correlations between protoporphyrin IX levels and QoL scores (Table S6). Moreover, there was no difference in QoL scores when comparing different FECH variant types (S7).

## 4 DISCUSSION

In this study we demonstrate a clinically relevant reduction in the QoL in children with EPP when compared to healthy children and adults with EPP. Within the QoL domains of the PedsQL, both physical and social functioning seem to be affected. When compared to adults with EPP, overall scores and disease severity scores were similar, however, the QoL scores were lower in children. Children with EPP exhibited a higher percentage of social inhibition compared to healthy children.

Quality of life in children with EPP was especially affected in the social and physical domains. The higher occurrence of social inhibition confirms the suspected impact of disease on social well‐being in these children. These findings are in line with prior research in adults with EPP that also reported a negative impact on social and physical functioning.^3^

The decreased physical functioning seems to result from pain, lower energy levels, and reduced sports participation. The painful phototoxic reactions characterizing the disease are likely to have a substantial impact on physical functioning. Previous studies have shown that EPP can affect the circadian rhythm, with later bedtimes and lower sleep efficiency in untreated adult patients.^13^ It is plausible that sleep is also affected in children with EPP, which could possibly explain the lower scores on energy levels. Other potential explanations may include anaemia^14^ and vitamin D deficiency,^15^, ^16^ often seen in EPP patients, or inflammation resulting from phototoxic reactions.^17^ The inability to participate in sports within the physical functioning domain, overlaps with the shortcomings patients experience in their social functioning, namely social participation. It is highly likely that sun‐avoiding behavior to prevent phototoxic reactions impacts the ability to participate in social activities, considerably impacting their QoL. Interestingly, teenagers with EPP seem to score higher in their physical functioning compared to children, suggesting that a better understanding and coping mechanisms may contribute to improved physical functioning.

When evaluating physical functioning of children with EPP in relation to children with other prevalent chronic diseases (e.g. diabetes and asthma), the EPP population exhibits higher scores.^18^ However, in the context of social functioning, EPP children demonstrate seemingly lower scores compared to children with other chronic diseases including diabetes, gastrointestinal conditions, and rheumatological disorders.^18^

Although children with EPP reported being restricted in school, needing adjustments, missing school because of hospital visits, and illness related to EPP, their overall school functioning was not affected. A previous study observing higher educational attainment in adults with EPP compared to the healthy population supports adequate school functioning in this population.^3^ That EPP does not affect school functioning could be explained by the children's lives being confined indoors, which could lead to improved school performance because they engage in more reading and learning activities. Although plausible, further research is needed to confirm this hypothesis.

Disease specific quality of life (EPP‐QoL) scores in children with EPP are found to be comparable to adults with EPP prior to treatment. However, when observing the subdomains a different pattern became evident. The matched adults scored similarly in the disease severity domain, but the QoL domain in children was substantially lower. This finding is in line with our hypothesis and what was previously suggested in other studies.^3^, ^7^ A possible explanation could be that as patients age, they develop an ability to cope with limitations associated with EPP and have adapted their lifestyle.^7^, ^19^ Despite this, adults still perceive their lives as significantly restricted.^7^, ^19^ Another reason could be the limited understanding the children and their family, friends and teachers have of their disease, coupled with a lack of age‐appropriate information.^7^

The impact of EPP on the QoL and social engagements of affected children underlines the importance for effective treatment options. Previous studies have shown up to 30% substantial improvement in EPP‐QoL in adult patients treated with afamelanotide,^3^, ^6^ Given the comparatively lower QoL among untreated children, similar, or potentially even better, results can be expected in children with effective treatment. However, the current available treatment (afamelanotide) has not yet received approval for children because of the absence of safety and efficacy data for individuals aged 18 and younger. Although afamelanotide is administered through subcutaneous implants, this mode may not be ideal for pediatric patients because of their invasiveness, alternatives such as bitopertin^20^ and dersimelagon,^21^ currently undergoing phase II‐III trials, are taken orally. We encourage the inclusion of children in trials to make treatment accessible for EPP patients across all age groups in the future.

Limitations of this study include the relatively small samples after stratification, making some comparisons difficult. The years of inclusion, consisting of years of lockdown due to the COVID‐19 pandemic, might also have affected the results. Healthy controls filled in questionnaires at the same time‐period, justifying this comparison. No significant differences in PedsQL scores during the pandemic could be found on stratification analysis (see Supporting Information). Sex could be a confounding factor, although previous studies on PedsQL in children found that boys and girls report similar QoL at a young age with no differences found in the social functioning domain.^22^, ^23^ Physical functioning could be affected in teenage girls, although physical functioning was also significantly lower in younger children with EPP, making confounding unlikely. We should, however, take into account that these variables might impact the reported QoL. Selection bias may be present as children with more severe EPP cases and a higher disease burden might be more inclined to attend doctor visits and thus be recruited for study participation. Despite these limitations, our results point towards a significantly reduced QoL in the children with EPP.

Strengths of this study lie in the relatively large sample of children with EPP, given the rarity of the disease, and the multicenter design with inclusion of two Northern European countries. Questionnaires from children and healthy controls were taken within the months EPP patients are most affected, namely spring and summer, mitigating the affect Northern European weather might have on the quality of life. This was, however, not taken into account for the questionnaires taken from the adults with EPP. Furthermore, the availability of data on adults with EPP enabled comparisons with both healthy controls and adults. This, and utilizing both general and disease‐specific QoL assessments, led to a comprehensive and thorough analysis of the QoL in children with EPP, thereby filling in an existing knowledge gap in the field of EPP. Although the EPP‐QoL questionnaire is not validated for children, our results suggest that this questionnaire is also appropriate in children with EPP.

In conclusion, children with EPP suffer from a markedly reduced QoL compared to both matched healthy controls and adults with EPP. Ensuring treatment availability for children with EPP is crucial for improving their QoL, particularly in terms of social and physical functioning. We advocate the inclusion of children and teenagers in safety and efficacy studies, for both new and existing drugs for EPP, to ensure availability of treatment in the future.

## FUNDING INFORMATION

The project and salary of the PhD candidate was funded by the department. There were no contracts related to this topic, nor were there any paid consultancies.

## CONFLICT OF INTEREST STATEMENT

Margreet Wagenmakers participates in contract studies for clinical trials with Ultragenyx Pharmaceutical, and Moderna (paid to institution). Peter Witters was funded by the Fonds Wetenschappelijk Onderzoek‐Vlaanderen (Fundamenteel Klinisch Mandaat 18B4322N). Janneke Langendonk participates in contract studies for clinical trials with Clinuvel Pharmaceuticals Ltd (producer of afamelanotide), Ultragenyx Pharmaceutical, and Alnylam® Pharmaceuticals (paid to institution). All other authors have no conflicts of interest to declare.

## INFORMED CONSENT

All subjects gave written informed consent for inclusion before they participated in the study. For the subjects aged 16 or younger, parents or legal guardians also gave written informed consent.

## ETHICS STATEMENT

The study was conducted in accordance with the Declaration of Helsinki. The study protocol was approved by the Ethics Committee of Erasmus Medical Center (MEC‐2020‐0706). The study protocol for the biobank including adults with EPP was also approved by the Ethics Committee of Erasmus Medical Center (MEC 2011‐525).

## Supporting information


Appendix S1.

## Data Availability

The data that support the findings of this study are available on request from the corresponding author(JGL). The data are not publicly available because they contain information that could compromise participants privacy.
