# Supplementary material for: Quality of life in children with erythropoietic protoporphyria: a case–control study
Source: J Dermatol. 2024 Jun 26;51(8):1068–78. doi: 10.1111/1346-8138.17348 (PMC11484138; doi:10.1111/1346-8138.17348)
Supplement: Supplementary file 1 — Appendix S1. [file JDE-51--s001.zip › jde17348-sup-0001-Figure S1 and Tables S1-S7.docx]

**Supplements**

## Figure S1: negative affectivity and social inhibitions scores of children with EPP (cases) versus healthy children (controls)


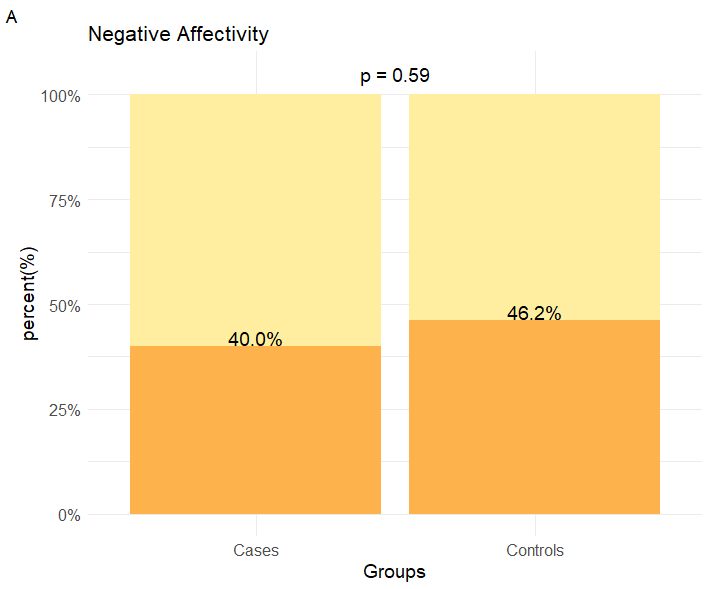


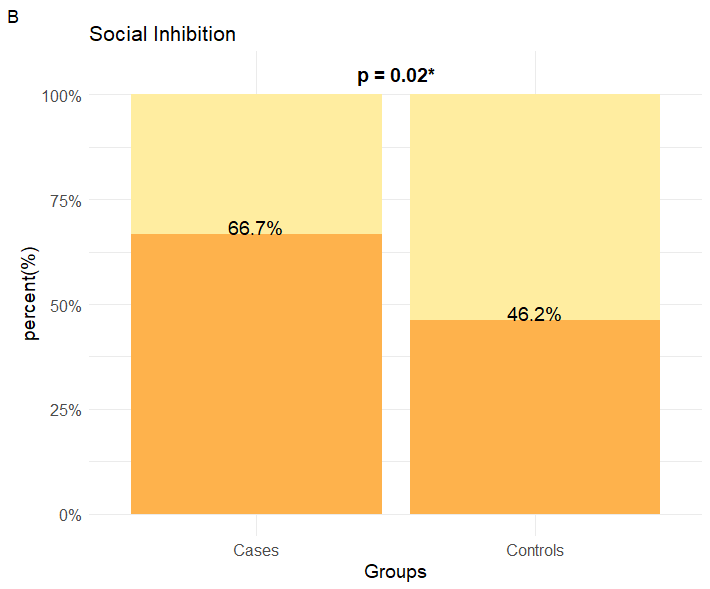


*The figure illustrate the percentage of children with negative affectivity or social inhibition from the DS-14 questionnaire. Children with EPP (cases) , are compared to matched healthy children (controls). The percentage is indicated within the figure, with p-values from the Chi-Squared test included. Significant p-values are indicated in bold and marked with an asterisk (*). The figure presents data for two categories: A) Negative Affectivity, and B) Social Inhibition.*

**Table S1: Paired analysis of the Quality of life based on Pediatric Quality of Life Inventory (PedsQL) scores of children with EPP and matched healthy children**

| Variable | Cases | Controls | P-value* |
| --- | --- | --- | --- |
| Participants (n) | 10 | 10 |  |
| Physical Functioning | 83.6 (77.0-90.2) | 98.4 (94.1-100.0) | 0.06 |
| Walking | 93.8 (78.1-100.0) | 100.0 (100.0-100.0) | 0.16 |
| Running | 100.0 (87.5-100.0) | 100.0 (100.0-100.0) | 0.41 |
| Sports Participation | 75.0 (56.3-100.0) | 100.0 (100.0-100.0) | 0.06 |
| Lifting heavy | 100.0 (78.1-100.0) | 100.0 (100.0-100.0) | 0.78 |
| Showering by myself | 100.0 (100.0-100.0 | 100.0 (100.0-100.0) | 1.00 |
| Chores around the house | 100.0 (81.3-100.0) | 100.0 (100.0-100.0) | 0.58 |
| Pain, Wounds | 56.3 (50.0-75.0) | 100.0 (75.0-100.0) | **0.02** |
| Low energy | 75.0 (65.6-84.4) | 100.0 (87.5-100.0) | **0.03** |
| Emotional Functioning | 75.0 (66.3-79.4) | 72.5 (58.1-94.4) | 0.81 |
| Afraid/ scared | 87.5 (75.0-87.5) | 75.0 (62.5-96.9) | 0.48 |
| Sad/ blue | 62.5 (62.5-96.9) | 62.5 (62.5 -96.9) | 1.00 |
| Angry | 75.0-40.6-81.3) | 75.0 (62.5-93.8) | 0.89 |
| Trouble sleeping | 56.3 (40.6-81.3) | 81.3 (53.1 -96.9) | 0.21 |
| Worrying | 81.3 (65.6-87.5) | 75.0 (62.5-96.9) | 0.90 |
| Social Functioning | 71.3 (68.1-84.4) | 100.0 (89.4-100.0) | 0.05 |
| Getting along with kids | 87.5 (75.0 -100.0) | 100.0 (87.5-100.0) | 0.53 |
| Kids wanting to be my friend | 100.0 (87.5-100.0) | 100.0 (87.5-100.0) | 0.79 |
| Teasing | 100 (100.0-100.0) | 100 (100.0-100.0) | 1.00 |
| Cannot do things other kids do | 75.0 (31.3-75.0) | 100.0 (90.6-100.0) | 0.06 |
| Hard to keep up when playing with other kids | 50.0 (37.5 -59.4) | 100.0(100.0-100.0) | **<0.01*** |
| School Functioning | 75.0 (72.5-85.0) | 86.3 (77.5-91.9) | 0.39 |
| Paying attention | 75.0 (62.5-100.0) | 87.5 (62.5-100.0) | 0.68 |
| Forgetting things | 75.0 (62.5-87.5) | 75.0 (65.6-84.4) | 0.41 |
| Keeping up with school work | 100.0 (75.0-96.9) | 81.3 (75.0-100.0) | 0.72 |
| Missing school | 75.0 (75.0-100.0) | 93.8 (75.0-100.0) | 0.67 |
| Missing school due to hospital | 75.0 (62.5-75.0) | 100.0 (90.6-100.0) | **0.04** |
| Psychosocial Score ^†^ | 73.3 (66.7-81.1) | 82.9 (76.7-94.6) | 0.36 |
| Total Score ^††^ | 77.5 (70.9-82.2) | 88.9 (83.8-96.1) | 0.09 |

*All data is given in median (interquartile range).* Scores range from 0 to 100, with higher scores indicating a higher QoL.

*P-values are calculated Wilcoxon signed rank test. All p-values <0.05 are bold, and all significant p-values after Bonferroni correction (0.05/4=0.0125 are noted by an asterisk (*)).*

^†^*includes emotional, social and school functioning*

^††^*includes physical, emotional, social and school functioning*

*Abbreviations. EPP, erythropoietic protoporphyria*

**Table S2: Quality of life based on Pediatric Quality of Life Inventory (PedsQL) Stratified by Age**

|  | Children (8-12 years) | Teenagers (13-18 years) | P-value |
| --- | --- | --- | --- |
| Children with EPP (Cases) | | |  |
| Participants (n) | 5 | 7 |  |
| Physical Functioning | 85.9 (14.1) | 89.1 (19.5) | 0.52 |
| Emotional Functioning | 72.5 (10.1) | 77.5 (26.3) | 0.42 |
| Social Functioning | 82.5 (15.0) | 72.5 (18.8) | 1.00 |
| School Functioning | 85.0 (12.5) | 77.5 (20.0) | 0.81 |
| Psychosocial Score^†^ | 82.5 (21.7) | 74.2 (23.3) | 0.42 |
| Total Score^††^ | 81.0 (13.6) | 79.3 (22.0) | 0.63 |
| Matched healthy children (Controls) | | |  |
| Participants (n) | 8 | 4 |  |
| Physical Functioning | 100.0 (1.6) | 84.5 (10.2) | 0.12 |
| Emotional Functioning | 71.3 (23.1) | 78.8 (20.6) | 0.67 |
| Social Functioning | 100.0 (14.4) | 78.8 (35.0) | 0.20 |
| School Functioning | 86.3 (13.1) | 71.3 (31.3) | 0.44 |
| Psychosocial Score^†^ | 80.4 (10.8) | 76.3 (29.0) | 0.81 |
| Total Score^††^ | 86.4 (7.6) | 82.6 (22.4) | 0.81 |

*Data is given in median and interquartile range. p-values from Mann-Whitney U tests included. Significant scores are denoted with an asterisk (*). ^†^ includes emotional, social and school functioning. ^††^includes physical, emotional, social and school functioning. Abbreviations. EPP, erythropoietic protoporphyria*

**Table S3: Quality of life based on Pediatric Quality of Life Inventory (PedsQL) Stratified by Biological Sex**

|  | Girls | Boys | P-value |
| --- | --- | --- | --- |
| Children with EPP (Cases) | | |  |
| Participants (n) | 10 | 2 |  |
| Physical Functioning | 87.5 (15.2) | 82.0 (16.4) | 0.75 |
| Emotional Functioning | 77.5 (13.1) | 82.5 (17.5) | 0.75 |
| Social Functioning | 77.5 (15.0) | 76.3 (13.8) | 1.00 |
| School Functioning | 81.3 (20.0) | 81.3 (8.8) | 1.00 |
| Psychosocial Score^†^ | 78.3 (18.3) | 80.0 (13.3) | 0.83 |
| Total Score^††^ | 80.2 (13.9) | 80.7 (14.4) | 1.00 |
| Matched healthy children (Controls) | | |  |
| Participants (n) | 6 | 6 |  |
| Physical Functioning | 96.9 (4.3) | 100.0 (0.0) | 0.12 |
| Emotional Functioning | 72.5 (18.8) | 73.8 (33.8) | 0.81 |
| Social Functioning | 97.5 (10.6) | 90.0 (23.8) | 0.73 |
| School Functioning | 81.3 (13.1) | 86.3 (23.1) | 1.00 |
| Psychosocial Score^†^ | 81.7 (10.6) | 79.2 (19.4) | 0.98 |
| Total Score^††^ | 87.0 (6.7) | 86.4 (12.6) | 1.00 |

*Data is given in median and interquartile range. p-values from Mann-Whitney U tests included. Significant scores are denoted with an asterisk (*). ^†^ includes emotional, social and school functioning. ^††^includes physical, emotional, social and school functioning. Abbreviations. EPP, erythropoietic protoporphyria*

**Table S4: Quality of life based on Pediatric Quality of Life Inventory (PedsQL) Stratified by Year of Inclusion**

|  | 2020 | 2022 | P-value |
| --- | --- | --- | --- |
| Children with EPP (Cases) | | |  |
| Participants (n) | 3 | 9 |  |
| Physical Functioning | 78.1 (12.5) | 89.1 (17.2) | 0.27 |
| Emotional Functioning | 65.0 (7.5) | 80.0 (20.0) | 0.06 |
| Social Functioning | 67.5 (10.0) | 85.0 (20.0) | 0.19 |
| School Functioning | 77.5 (11.3) | 85.0 (17.5) | 0.92 |
| Psychosocial Score^†^ | 66.7 (7.9) | 83.3 (20.0) | 0.35 |
| Total Score^††^ | 70.7 (9.5) | 81.0 (19.6) | 0.27 |
| Matched healthy children (Controls) | | |  |
| Participants (n) | 3 | 9 |  |
| Physical Functioning | 95.3 (3.1) | 100.0 (1.6) | 0.55 |
| Emotional Functioning | 85.0 (13.8) | 70.0 (20.0) | 0.19 |
| Social Functioning | 95.0 (6.3) | 100.0 (25.0) | 0.92 |
| School Functioning | 85.0 (6.3) | 85.0 (32.5) | 1.00 |
| Psychosocial Score^†^ | 88.3 (8.8) | 76.7 (11.7) | 0.37 |
| Total Score^††^ | 90.8 (6.8) | 84.8 (7.6) | 0.48 |

*Data is given in median and interquartile range. p-values from Mann-Whitney U tests included. Significant scores are denoted with an asterisk (*). ^†^ includes emotional, social and school functioning. ^††^includes physical, emotional, social and school functioning. Abbreviations. EPP, erythropoietic protoporphyria*

**Table S5: Quality of life based on Pediatric Quality of Life Inventory (PedsQL) comparing child and parent-proxy reports**

|  | Children with EPP (Cases) | | | Matched healthy children (Controls) | | |
| --- | --- | --- | --- | --- | --- | --- |
|  | **Child Self-reported** | **Parent Proxy-report** | **P-value*** | **Child Self-reported** | **Parent Proxy-report** | **P-value*** |
| Participants (n) | 13 | 12 |  | 12 | 12 |  |
| Physical Functioning | 90.6 (20.3) | 85.9 (13.3) | 1.00 | 98.4 (4.7) | 100.0 (0.0) | 0.17 |
| Emotional Functioning | 85.0 (17.5) | 72.5 (16.3) | 0.70 | 72.5 (21.3) | 70.0 (21.3) | 0.90 |
| Social Functioning | 85.0 (23.8) | 72.5 (22.5) | 0.08 | 95.0 (18.8) | 100.0 (45.0) | 0.002* |
| School Functioning | 75.0 (22.5) | 80.0 (15.0) | 0.74 | 80.0 (18.8) | 87.5 (16.3) | 0.91 |
| Psychosocial Score^†^ | 78.3 (22.5) | 76.7 (19.2) | 0.48 | 79.2 (16.3) | 79.2 (20.0) | 0.95 |
| Total Score^††^ | 81.5 (14.9) | 77.7 (15.0) | 0.57 | 84.8 (11.4) | 86.4 (15.5) | 0.79 |

*Data is given in median and interquartile range. p-values from Mann-Whitney U tests included. Significant scores are denoted with an asterisk (*). ^†^ includes emotional, social and school functioning. ^††^includes physical, emotional, social and school functioning. Abbreviations. EPP, erythropoietic protoporphyria*

**Table S6: correlation between protoporphyrin IX in erythrocytes and quality of life scores in children with EPP**

| Variable 1 | Variable 2: Quality of life | Number of participants (n) | Spearman’s rho | P-value* |
| --- | --- | --- | --- | --- |
| Protoporphyrin IX (µmol/L erythrocytes) | EPP-QoL: total score (%) | 13 | -0.11 | 0.72 |
|  | EPP-QoL: severity of disease domain (%) | 13 | -0.12 | 0.68 |
|  | EPP-QoL: quality of life domain (%) | 13 | 0.07 | 0.81 |
|  | PedsQL: physical functioning (%) | 11 | -0.34 | 0.31 |
|  | PedsQL: emotional functioning (%) | 11 | -0.16 | 0.63 |
|  | PedsQL: social functioning (%) | 11 | -0.01 | 0.97 |
|  | PedsQL: school functioning (%) | 11 | -0.09 | 0.78 |
|  | PedsQL: psychosocial score^†^ (%) | 11 | -0.11 | 0.74 |
|  | PedsQL: total score^††^ (%) | 11 | -0.29 | 0.38 |

**p-values from Spearman’s correlation test are included. Significant scores are denoted with an asterisk (*).^†^ includes emotional, social and school functioning. ^††^includes physical, emotional, social and school functioning. Abbreviations. EPP, erythropoietic protoporphyria; EPP-QoL, EPP quality of life ; PedsQL, pediatric quality of life inventory*

**Table S7: Quality of life scores in children with EPP stratified by FECH variants type**

|  | Missense | Splice-site | Deletion | Deletion/ insertion |
| --- | --- | --- | --- | --- |
| Participants (n) | 7 | 5 | 1 | 1 |
| EPP-QoL: total score (%) | 47.2 (34.7-40.9) | 41.7 (25.0-58.3) | 80.6 | 25.0 |
| EPP-QoL: severity of disease domain (%) | 50.0 (38.3-55.0) | 50.0 (26.7-60.0) | 90.0 | 30.0 |
| EPP-QoL: quality of life domain (%) | 16.7 (8.3-25.0) | 16.7 (0.0-33.3) | 33.3 | 0.0 |
| PedsQL: physical functioning (%) | 85.9 (78.1-89.1) | 81.3 (73.4 -98.4) | 100.0 | 90.6 |
| PedsQL: emotional functioning (%) | 72.5 (55.0-77.5) | 77.5 (65.0-97.5) | 97.5 | 70.0 |
| PedsQL: social functioning (%) | 67.5 (52.5-72.5) | 70.0 (70.-85.0) | 90.0 | 82.5 |
| PedsQL: school functioning (%) | 72.5 (70.0-77.5) | 72.5 (72.5-90.0) | 97.5 | 95.0 |
| PedsQL: psychosocial score^†^ (%) | 66.7 (59.2-74.2) | 72.5 (66.7-92.5) | 95.0 | 82.5 |
| PedsQL: total score^††^ (%) | 71.7 (70.6-79.4) | 75.5 (66.3-95.1) | 96.7 | 85.3 |

*Data is given in median and interquartile range.^†^ includes emotional, social and school functioning. ^††^includes physical, emotional, social and school functioning. Abbreviations. EPP, erythropoietic protoporphyria; FECH, ferrochelatase; EPP-QoL, EPP quality of life ; PedsQL, pediatric quality of life inventory*
